# Demand and Financial Constraints in Eliminating Architectural and Technical Barriers for People with Disabilities in Poland

**DOI:** 10.1155/2018/1297396

**Published:** 2018-02-19

**Authors:** Maria Hełdak, Agnieszka Stacherzak, Katarzyna Przybyła

**Affiliations:** Department of Spatial Economy, Wroclaw University of Environmental and Life Sciences, Wrocław, Poland

## Abstract

The purpose of the study is to analyse the availability of financial resources for people with disabilities and to assess the needs satisfaction level of the disabled in order to eliminate architectural and technical barriers in Poland. The research conducted among the disabled affected by physical disability indicates that mobility barriers and obstacles remain among the most important problems encountered by people with disabilities. The research has shown that the problem of barriers increases with age. The elimination of architectural barriers requires, each time, higher financial expenditure, whereas the elimination of technical barriers improves the life quality of people with disabilities at low financial outlays. The average funding in Poland amounted to PLN 827.53 in 2016, including the funding of EUR 1453.60 for the elimination of architectural barriers and approx. EUR 582 for the removal of technical barriers. The financial resources allocated for this purpose do not cover the actual needs of the people with disabilities. The analysis revealed that the demand for investment in the elimination of barriers is increasing with age, whereas the expenditure of the Polish state is decreasing.

## 1. Introduction

One of the main reasons for low social and spatial participation of the disabled is the presence of various barriers. They manifest themselves mainly in form of architectural barriers that physically prevent disabled persons from being mobile. However, the notion of barriers should be understood more broadly, as one of the main components of the perception of reality by people with disabilities. The existence of barriers, whether real or imaginary, in the awareness of the disabled is of major social importance.

According to Groessl et al. [1], disability is reflected in deteriorated quality of life. On the other hand, persons with disabilities are one of the social groups that are the most exposed to marginalisation [2]. The European Union Member States have been implementing measures to prevent poverty and social exclusion since 2001.

According to the results of the National Census of Population and Households of 2011, conducted by the Central Statistical Office, as of the end of March 2011 [3], the total number of people with disabilities in Poland was 4697 million (12.2% of the total population). According to the World Health Organization, one in every 10 people has a disability [4].

At the same time, the number of the biologically disabled was 4217.6. Biologically disabled persons were defined as those who feel limited in performing the usual (basic) daily life activities adequate to their age. In Poland, 3 degrees of limitation of the capacity to perform basic activities are distinguished: complete, serious, or moderate incapacity. In all these cases, the duration of actual or predicted inability to perform the discussed actions should be at least 6 months.

According to the results of the European Health Interview Survey (EHIS), in 2014, 7689.8 (7.7 million) biologically disabled persons, that is, people who declared limited capacity to perform the activities usually performed by people, lived in Poland [5]. Among those persons, 2464.8 had serious capacity limitations and 5225 complained of less serious limitations.

Architectural barriers mean any obstacles in the building and its nearest vicinity that prevent or hinder free mobility of the disabled as a result of technical solutions, construction, or the conditions of use.

The following authors wrote about the architectural and technical barriers for people with disabilities and their negative impact on self-esteem, the mobility of people suffering from physical disabilities, and also self-reliance in dealing with everyday activities: Siqueira et al. [6], Hasseler [7], and Meyer-Bohe et al. [8].

Disability affects three areas of lives of the concerned persons: their health functions, as well as social and psychological functions [9]. According to Bartkowski [10], the definition of disability reveals three possible ways to determine and measure the problem: (I) based on clinical variables (the aetiology, location, and scope of disorders), (II) the ability to perform daily activities (self-care activities, mobility), and (III) the ability to perform basic social roles adequate to the age of the individual.

The authorities responsible for solving the problems of disabled people by means of eliminating architectural barriers that prevent or hinder free mobility of such persons are the poviat-level authorities. The task is financed from the means of the State Fund for the Rehabilitation of Persons with Disabilities (PFRON). The body is responsible for the realisation of these tasks to enable or to facilitate, to a significant extent, the performance of basic daily activities or interactions with the environment by people with disabilities.

The purpose of the study is to analyse the availability of financial resources for people with disabilities and to assess the needs satisfaction level of the disabled in relation to the elimination of architectural and technical barriers in Poland.

The research has shown the scale of demand for the elimination of architectural and technical barriers and the capacity for meeting these needs from the state financial resources. They basically present the situation of people with disabilities and also the progress in the improvement of their living conditions from the architectural perspective.

## 2. Materials and Methods

The research work involved the following activities:

- The number of disabled persons in Poland was determined.
- The principles and financial expenditures of the State of Poland incurred on eliminating architectural and technical barriers were determined.
- The demand and number of filed applications for financing the elimination of architectural, technical, and communication barriers were determined.
- The financial capacity of the disabled in actions aimed at the elimination of transport barriers was specified.
- A detailed analysis of the related investments in a selected capital of the province in Poland was conducted.

Source data were obtained from the Central Statistical Office of Poland (GUS), the European Health Interview Survey (EHIS), and the Management of the State Fund for the Rehabilitation of Persons with Disabilities (PFRON). Then, a comparative analysis of the collected data was conducted.

The following abbreviations, derived from the names of Polish bodies and institutions, as well as the analyses generally applicable in Poland, were used in the study:

- BAEL—Survey of Economic Activity of the Population
- PFRON—The State Fund for the Rehabilitation of Persons with Disabilities
- EHIS—European Health Interview Survey
- GUS—Central Statistical Office

The paper should refer to the scale of the phenomenon—its reach and size. Such analysis is confronted with two essential issues already on the initial stage [11]. The first one is the number (various estimation criteria) and the other—the dynamics of the group of the disabled.

Functional criteria enable to evaluate the demand for social care benefits or the necessary technical aids. The analysis in terms of potential use in mass studies demonstrates that these criteria are difficult to be thoroughly analysed, as the group of the disabled is much larger than the group of people who have the relevant medical certificate (this applies mainly to the elderly).

For the purposes of the conducted research, a person with disability is considered the one having an adequate medical certificate about disability (referred to as the so-called legal disability).

The number determined in such a way is the lowest and it does not reflect the whole disabled population. However, the data are gathered continuously and they set up the basis for the information included in the annual government report presented to the Parliament. The Parliament—The Parliament is the first chamber (traditionally referred to as the lower chamber) of Polish parliament. It represents the legislative power in Poland. The basic function of the Parliament consists in enacting, by means of a legislative procedure, the legal acts—statutes deciding on the functioning rules in the basic areas of life covering the citizens.

## 3. The Number of Disabled Persons in Poland

According to the results of the BAEL [12], the number of the legally disabled in 2011 approached 3.4 million (precisely 3384). This included approx. 2024 legally disabled persons in working age, which accounts for 8.4% of this age group. This means that 10.6% of the population aged 15 and over is legally certified as disabled, that is, that every 10th adult in Poland is disabled. In the working age group, this refers to every 12th person.

At the same time, the BAEL data highlight that both the relative and absolute numbers of persons with disabilities are decreasing. During 14 years (1997–2011), the number of the legally disabled fell by 1.3 million and of the disabled in the working age group by approx. 220 thousand during 9 years (2002–2011).

However, the data concerning the number of disabled persons in Poland as revealed by the National Census are different [3]. According to censuses, although the number of the disabled in Poland diminished in the years 2002–2011, the data show an increase in their number, both in absolute terms and in terms of percentage of the population (Table 1).

In the period between the censuses of 1988 and 2002, the number of persons with disabilities increased by 1721.2 (i.e., by 46.1%). This number was reduced in comparison to the year 2002, when the number of the disabled determined pursuant to the National Census reached approx. 5.5 million (12.2% of the total population). Only a comparison in a period longer than 30 years—between 1978 and 2011—demonstrates that the percentage of the disabled increased from 7.1% to 12.2% of the total population [11, 13].

The studies carried out worldwide confirm an ongoing increase in the number of people with disabilities in the group of senior citizens—over 70 years of age [14]. The similar situation was recorded in Western and Central European countries. Research conducted in Germany on a large group of elderly persons (*n* = 4117) demonstrated that nearly every second respondent suffered from disability (44.7%). The problems concerned in particular women, people with low income, and those suffering from joint or eye dysfunctions [15].

In the year 2060, almost 25% of the Swedish population is expected to be over 65 years old, as compared to 19% in 2011. The situation is similar in many Western countries [16].

In Poland, a significant share of the disabled in the group of people aged over 70 years has also been noted (Tables 2 and 3).

Unfortunately, no current analyses are available for the specified age groups. The only results available refer to persons under the age of 16 and according to working and postworking age groups. In 2011, the group of disabled persons in the postworking age group in Poland increased to 40% in comparison to the elderly disabled persons in 2002.

According to the findings of EHIS, in 2014, depending on the adopted biological disability criteria (or, more precisely, the impairment degrees), the population of persons with disabilities in Poland may range from 4.9 to 7.7 million people. According to methodology currently used in health condition analyses, the population of disabled persons was 4.9 million. This number included both persons who had a legal disability certificate and (and/or) those with limited capacity to perform activities, but only to a serious degree.

The data on the numbers of the disabled vary. The comparison of data from various sources reveals different numbers. Differences exist even between basic sources, such as the national census, data on the economic activity of the population, and the health condition analysis. These differences may reach even millions of people.

## 4. Demand for Works Connected with Eliminating Architectural Barriers

The results of previous research on the disabled revealed high importance of various types of barriers in the daily life of persons with disabilities. Studies among the disabled often demonstrate that such barriers or obstacles that hinder mobility and daily life activities or errands are ranked high among their problems. The mobility of adults with disabilities in 1996, according to age groups, is presented below (Table 4).

These studies have demonstrated that the problem of barriers becomes more significant with increasing age. In older age groups, the living space of the disabled becomes increasingly limited to their homes and the nearest neighbourhood. Although a lot has been done to improve the situation of the disabled since that time, one can still assume that the results show how restricted the space accessible for the disabled is, especially for the elderly ones.

At the same time, in this age group, the overall health deteriorates and its members often suffer from more than one illness. The elderly belong to the social group with considerably lower income and resources. Their possibility to satisfy their needs is reduced and they are often threatened by poverty. For this group, receiving assistance in meeting daily needs is of high importance, while various types of barriers become a major hindrance, which is felt much more strongly than on the earlier stages of life. These barriers reduce mobility and limit the possibilities of social participation. Thus, social inclusion requires a series of measures in form of material support, care, and assistance in satisfying daily life needs. In this age group, the policy of meeting needs related to “quality of life” may be of great importance.

The main problem faced by persons with disabilities in Poland (although not only here) is the risk of impoverishment and the fact that their families actually slide into permanent poverty, which leads to a series of further negative consequences for their social relationships, psychological condition, the ability to actively participate in community life, and, last but not least, health [13]. In fact, the disabled can rarely afford to adapt their apartments to suit their special needs and to build ramps or other improvements in the vicinity of their homes.

The average monthly pension and disability benefit paid by the Social Security Office, according to the type of benefit, is the equivalent of approx. 44% of the average remuneration and in 2017 it equals approx. EUR 435.00 (PLN 1848.00). This amount is insufficient to satisfy the basic daily needs and to pay the utility bills. The financial standing of disabled persons is strongly affected by their family situation. There is a considerable difference between persons who live alone and those who live in family households, regardless of their size. The latter enjoy much better material situation.

There is a very low rate of employment of the disabled in Poland caused not only by their obvious physical condition but also architectural barriers and totally unsuitable means of public transport they cannot bear [18].

Merely equipping the homes with additional facilities for the disabled is highly insufficient. Although a majority of persons with disabilities declare that it is not necessary to adapt their homes to the limitations resulting from disability (Table 5), the absence of such amenities in the homes of the remaining group is a major problem.

It was determined that persons with serious mobility dysfunctions and blind people, who require lifts, other lifting equipment, adapting bathrooms, nonslip floors, or special texture floors, are in the worst situation [19].

## 5. Principles of Financing the Elimination of Architectural Barriers in Poland

The main organisation that provides financial support for investments aimed at eliminating mobility barriers in Poland is the National Disabled Persons Rehabilitation Fund, financed from the state budget. Acting pursuant to the Act of 27 August 1997 on Vocational and Social Rehabilitation and Employment of Persons with Disabilities, PFRON, Art. 47, considers it justified to fund, as part of the elimination of architectural, town-planning, and communication barriers, certain investments in order to enable persons with disabilities to participate in community life.

In order to enable the realisation of the relevant scopes of works that allow the disabled to function independently in the society, the following types of disability were distinguished:

- Persons who move with the use of a wheelchair
- Persons with reduced mobility—others not listed in item (I)
- Persons with sight dysfunctions
- Persons with hearing or speech dysfunctions
- Persons with other disabilities

In Poland, the investments that may be financed from the state budget are divided into five groups, according to the type and degree of disability (Table 6).

At the same time, in order to receive financing, the applicant has to meet certain criteria. Basic (selected) principles of financing the investment in eliminating barriers are presented below:

1. The amount of financing of the elimination of architectural barriers may reach up to 95% of the investment value, although not more than the equivalent of fifteen times the average monthly wage. The financing amount is determined on an individual basis.
2. Financing from the means of the fund may be granted for the purchase of equipment, construction materials, works, or other activities that may be financed as part of eliminating architectural barriers.
3. Upon the qualification of the application for realisation, the applicant shall submit
  - a construction design (drawing of the premises before and after modernisation),
  - a preliminary cost estimate or pricing.
4. All construction works should be performed in compliance with the provisions of the Act of July 7, 1994—Construction Law [21] and the Ordinance of the Minister of Infrastructure of April 12, 2002, on the technical conditions to be met by buildings and their location [22].

## 6. Amount of State Financing of the Elimination of Architectural and Technical Barriers in Poland

Pursuant to the Ordinance of the Minister of Labour and Social Policy of June 25, 2002, the duty of financing the elimination of architectural, communication, and technical barriers is vested in poviats. Architectural barriers are defined as any obstacles that exist in the building and its nearest vicinity that prevent or hinder free mobility of the disabled due to technical solutions, construction, or the conditions of use. On the other hand, technical barriers are those that result from the lack of application or failure to introduce adaptations of premises or equipment adequately to the type of disability.

The table below presents the amount of the total financial aid granted to persons with disabilities in Poland (Table 7).

**Figure 1:**
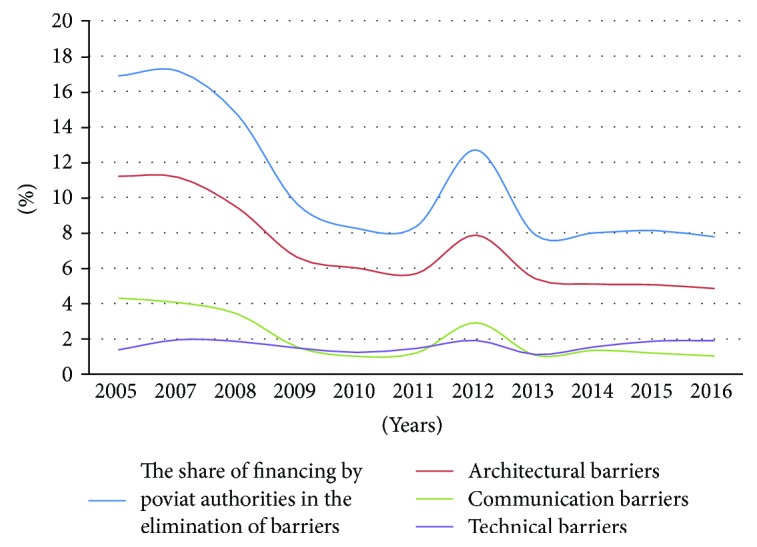
The share of financing by poviat authorities in the elimination of architectural, technical, and communication barriers in the years 2005–2016. Source: own study based on annual reports published by PFRON.

The share of financing the elimination of architectural, technical, and communication barriers on request of individuals in total financial aid granted to the disabled by poviat self-government authorities in Poland is presented in Figure 1.

**Figure 2:**
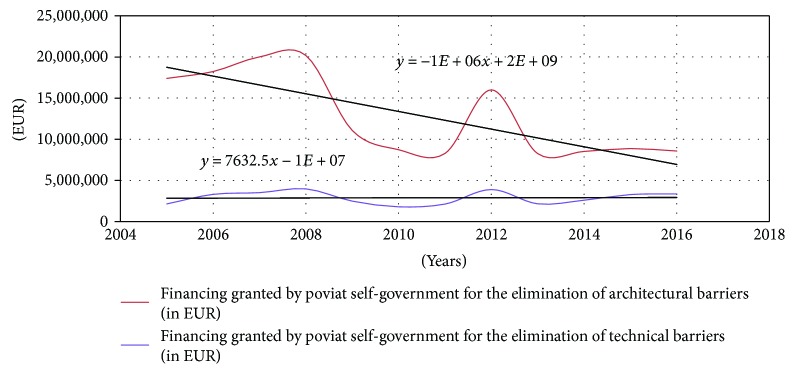
Financing granted by poviat self-government authorities for the elimination of architectural and technical barriers in the years 2005–2016. Source: own study based on annual reports published by PFRON.

Unfortunately, the share of funds granted for the elimination of disadvantages faced by the disabled in their environment in the yeas 2005–2016 was characterised by a decreasing trend. Only the funding granted for the removal of technological barriers remained stable. Eliminating these barriers improves the activity of the disabled persons in the community and allows them to function more efficiently. Currently, the funds of poviat self-government authorities are being allocated to financing the supplies of orthopaedic aids, rehabilitation, and supporting economic activity. The decreasing trend in granted financing in the analysed period refers mainly to the correction of architectural barriers (Figure 2).

The elimination of architectural barriers always requires considerable financial expenditures, while the removal of technical barriers improves the quality of life of the disabled at a relatively low expense. Funding is granted if it is justified by the needs resulting from disability.

The interest in receiving financial aid is very high, yet the available funds are limited. Average amounts of financing of investments realised in the years 2011–2016 are listed below (Table 8).

**Figure 3:**
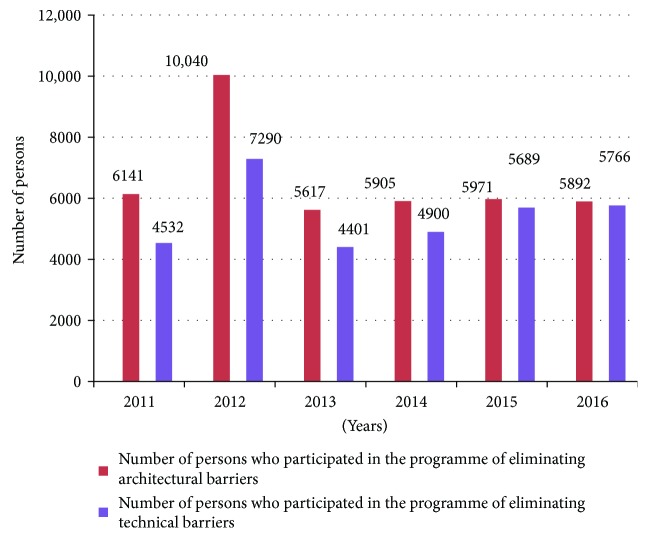
Number of persons with disabilities who participated in the programme of eliminating architectural and technical barriers in the years 2011–2016. Source: own study based on annual reports published by PFRON.

The number of persons with disabilities who participated in the programme pursuant to the applications realised in the years 2011–2016 is listed below (Figure 3).

Nearly all poviats participated in the realisation of tasks of the poviat self-government authorities financed by PFRON (in 2015–374 poviats, that is, 98.42% of all poviats in Poland). Nearly 16,000 persons take part in the programme annually (17,130 persons with disabilities in 2015, 16,605 persons in 2016).

Due to the extremely low level of pension and disability benefits paid to the disabled in Poland, persons with mobility disorders are unable to realise the investments at their own expense.

## 7. Conclusions

The conducted research allowed the authors to formulate the following conclusions:

1. The funding aimed at the elimination of architectural barriers is intended only for people with disabilities, presenting a medical certificate of their disability. Only such individuals may apply to receive funding for construction work or changes in interior finishing due to mobility disability.
2. The analysis revealed a constant increase in the number of disabled persons in the postworking age group, which results to a growing need to provide care and assistance to the elderly (who accounted for approx. 40% of the total number of disabled persons in Poland in 2011).
3. The group of elderly persons includes a considerable group of formally “healthy” persons (without the relevant medical certificates), who, however, face significant health-related limitations. These people are, to a large extent, only “biologically” disabled. Social policy should pay attention to their needs and develop instruments to support them.
4. Taking into account the low level of state pensions and annuities received by the disabled, it is indispensable to support them in adjusting their apartments/houses or to provide mobility facilities for the needs of people with reduced mobility. The demand for this type of construction work is continuously increasing due to the growing share of people over 70 years of age.
5. Polish government recognises the need to solve the problems of disabled people by means of eliminating architectural barriers that prevent or hinder free mobility of such persons. Their funding is, however, insufficient.
6. The financial means allocated to supporting persons with disabilities in eliminating architectural and technical barriers are limited. They account for approx. 8% of the expenditures of poviat authorities on persons entitled to receive aid. Funds actually granted in 2016 are lower by half than the subsidies granted in 2005.
7. The total financing granted for the elimination of technical barriers remains stable, although the individual amounts of funding paid have increased. These investments improve the mobility of the disabled in their environment, while requiring relatively low amounts of funding.

## Figures and Tables

**Table 1:** Disabled persons in the social structure according to the national censuses in the years 1978, 1988, 2002, and 2011 (in thousands).

Year of the census	Disabled persons in the year			
	1978 (thousands)	1988 (thousands)	2002 (thousands)	2011 (thousands)
Total number of disabled persons	2485.0	3735.5	5456.7	4697.5
Legally disabled persons	1928.6	3258.4	4450.1	3131.9
Biologically disabled persons	556.4	477.1	1006.6	1565.6
Disabled persons—per 1000 residents	71	99	143	122

Source: GUS.

**Table 2:** Disabled persons according to the main age groups in the years 2004 and 2009.

Age	Disabled persons			
	Year 2004		Year 2009	
	(Thousands)	%	(Thousands)	%
Total	6205.9	100	5258.2	100
0–14 lat	257.6	4.2	179.7	3.4
15–29 lat	390.4	6.3	329.3	6.3
30–49 lat	1061.3	17.1	746.6	14.2
50–69 lat	2741.0	44.2	2316.3	44.1
70 and over	1804.3	29.1	1686.3	32.1

Source: GUS.

**Table 3:** Disabled persons according to the main age groups in the years 2002 and 2011.

Age	Disabled persons			
	Year 2002		Year 2011	
	(Thousands)	%	(Thousands)	%
Total—legally disabled persons	4450	100	4697	100
Including those aged under 16	135	3	184.80	4.5
Aged 16 and over	4315	97.0	4512.2	95.5
Legally disabled persons in the working age group (18–59/64)	2608	58.6	2282.5	54.3
Legally disabled persons in the postworking age group	1700	38.2	2189.7	40.40

Source: GUS.

**Table 4:** Mobility of adults with disabilities in 1996 according to share (%).

Age group	Persons with limited mobility	Persons whose living space is limited		
		To their beds	To their homes	To their homes and neighbourhood
	As % of the particular age group			
50–54	7.5	0.2	1.5	5.8
55–59	10.9	0.6	1.4	8.9
60–64	14.9	0.7	2.8	11.3
65–69	20.3	1.5	4.6	14.3
70–74	25.5	1.0	5.6	18.9
75–79	32.7	3.4	6.4	23.0
80 and over	52.9	8.2	15.5	29.2

Source: Frąckiewicz [17].

**Table 5:** Level of satisfaction of needs in terms of the presence of amenities and adaptations for the disabled at home (%).

Specification of investments	Percentage of persons who need them	Owners among those in need
Various handles/grippers	18	44
Adapted bathrooms/toilets	16	40
Nonslip floors	15	31
Additional handrails along the walls	14	39
Adapted thresholds in the floor	14	51
Ramps	9	43
Widened door frames	7	46
Lift	7	28
Floors of different texture/colour	5	24
Lifting equipment	4	18
None of the above	73	x

Source: own study based on reports published by PFRON.

**Table 6:** Substantial scope of investments that may be financed from the state budget according to the type and degree of disability.

Item	Disability degree	Substantial scopes qualified for financing
I	Persons who move with the use of a wheelchair	(1) Change of the apartment for another, of a similar surface area (from a higher floor to the ground floor) that requires little adaptation works, to be made available to the applicant, along with documented house moving costs. (2) Construction of ramps and access ways that allow the disabled person to leave and return home on their own. (3) Lifts, stair platforms, stair transporters, wall lifts, and other vertical transport equipment. (4) Adaptation and modernisation of the apartment, including the kitchen, toilet, and bathroom, to enable the disabled person to function independently. (5) Installing handrails along transport routes, in toilets, and in bathrooms at home. (6) Removal of thresholds and levelling the floors in different rooms. (7) Adapting the doorways to the needs of the applicant. (8) Adapting the window frames to be operated by the applicant who lives with another disabled person or on their own. (9) Nonslip floors. (10) Installing automatic garage doors for the applicant at the homes of disabled persons who live with another disabled person or on their own. (11) Adapting kitchen equipment to enable independent use, including (a) low countertops that allow to move the wheelchair under the surface, (b) installing power sockets and switches in an area accessible for a sitting person. (12) Installation of phone and fax.

II	Persons with reduced mobility—others not listed in item I	Having performed an assessment of the degree of independence at home for persons with serious and moderate disabilities, the representatives of PFRON decide, at their own discretion, which elements listed for group I are required to be financed.

III	Persons with sight dysfunction	(1) Removal of thresholds. (2) Nonslip floors. (3) Installing handrails and handle bars. (4) Installing sound alarm and signalling systems. (5) Application of colour and texture solutions to improve spatial orientations. (6) Installing gas or electric central heating systems at homes of disabled persons who live with another disabled person or on their own. (7) Installing landline phones for applicants with moderate or serious disability. (8) Replacement of coal and gas stoves with electric or microwave cookers. (9) Changing the lighting and additional lighting in rooms.

IV	Persons with hearing or speech dysfunctions	(1) Light signalling systems, for example (a) equipping the doorbell with a light signal system, (b) equipping the phone with a visual signal system. (2) Purchase and installation of a fax. (3) Purchase and installation of tele-loop units. (4) Purchase and installation of a telephone amplifier.

V	Persons with other disabilities	Having performed an inspection at home to assess the degree of independence for persons with serious and moderate disabilities, the Representatives of PFRON decide, at their own discretion, which elements listed for group I or deemed as necessary by the Representatives are required to be financed.

Source: own study based on the Act of 27 August 1997 on Vocational and Social Rehabilitation and Employment of Persons with Disabilities [20].

**Table 7:** The amount of the total financial aid granted to persons with disabilities in Poland in the years 2005–2011 (in EUR).

Year	Amount of financing the elimination of architectural, technical, and communication barriers on individual requests (EUR)	Amount of financing the elimination of barriers on individual requests (EUR)		
		On architectural barriers (EUR)	On communication barriers (EUR)	On technical barriers (EUR)
2005	26,207,680.24	17,389,928.89	6,675,440.33	2,143,135.77
2006	30,288,582.29	18,224,095.83	8,759,989.87	3,305,271.03
2007	30,798,699.82	20,006,487.56	7,282,703.45	3,511,792.23
2008	31,431,846.63	20,170,833.08	7,293,350.29	3,970,803.33
2009	16,107,038.92	11,064,822.70	2,562,092.40	2,481,383.28
2010	11,983,567.38	8,724,818.81	1,471,397.49	1,788,420.76
2011	12,146,301.07	8,272,372.37	1,750,176.83	2,124,705.91
2012	25,826,797.29	16,000,504.60	5,925,415.90	3,888,586.18
2013	12,047,032.81	8,253,590.30	1,633,262.84	2,154,228.28
2014	13,374,485.92	8,518,685.96	2,253,148.67	2,592,415.03
2015	14,246,006.64	8,871,412.81	2,088,896.73	3,279,791.34
2016	13,748,134.27	8,566,388.23	1,818,451.30	3,358,846.94

Source: own study based on annual reports published by PFRON.

**Table 8:** Average amount of financing the elimination of architectural, technical, and communication barriers in the years 2011–2016.

Year	Average amount of financing the elimination of architectural, technical, and communication barriers on individual requests (EUR)			
	Total average	On architectural barriers	On communication barriers	On technical barriers
2011	770.41	1345.39	344.49	467.95
2012	798.82	1593.37	395.75	533.09
2013	825.65	1468.92	357.87	489.37
2014	764.76	1442.28	338.12	528.88
2015	831.06	1485.31	381.62	576.37
2016	827.53	1453.60	367.53	582.29

Source: own study based on annual reports published by PFRON.

## Additional Points

*Explanations and Future Research*. In the course of the research, certain limitations were encountered, which referred to defining the actual number of people presenting the need to eliminate architectural and technical barriers. The analysis covering the health condition of senior citizens (in the postproduction age) should be conducted to determine the extent of such individuals' mobility limitations. The study should be supported by a survey carried out among people in the postproduction age. It will allow indicating the actual demand for construction work, as well as changes in interior finishing designed to remove mobility barriers. This part of the society rarely applies to be granted the status of a disabled person and, therefore, does not take advantage of the state funds for the elimination of architectural barriers.
